# Stacked nanocarbon photosensitizer for efficient blue light excited Eu(III) emission

**DOI:** 10.1038/s42004-019-0251-z

**Published:** 2020-01-03

**Authors:** Yuichi Kitagawa, Fumiya Suzue, Takayuki Nakanishi, Koji Fushimi, Tomohiro Seki, Hajime Ito, Yasuchika Hasegawa

**Affiliations:** 1 Faculty of Engineering, Hokkaido University, Kita-13, Nishi-8, Sapporo Hokkaido, 060-8628 Japan; 2 Institute for Chemical Reaction Design and Discovery (WPI-ICReDD), Hokkaido University, Sapporo Hokkaido, 001-0021 Japan; 3 Faculty of Industrial Science and Technology, Tokyo University of Science, 6-3-1, Niijuku, Katsushika-ku Tokyo, 125-8585 Japan

**Keywords:** Energy harvesting, Photochemistry, Organic-inorganic nanostructures

## Abstract

Photosensitizer design to allow effective use of low-energy light is important for developing photofunctional materials. Herein, we describe a rational photosensitizer design for effective use of low-energy light. The developed photosensitizer is a stacked nanocarbon based on a rigid polyaromatic framework, which allows efficient energy transfer from the low-energy T_1_ level to the energy acceptor. We prepared an Eu(III) complex consisting of a luminescent center (Eu(III)) and stacked-coronene photosensitizer. The brightness of photosensitized Eu(III) excited using low-energy light (450 nm) is more than five times higher than the maximum brightness of previously reported Eu(III) complexes.

## Abstract (web summary)

Rational photosensitizer design for the effective use of low-energy light is important for photofunctional materials. Here the authors develop a stacked nanocarbon photosensitizer based on a rigid polyaromatic framework, which allows efficient energy transfer to a Eu(III) center.

## Introduction

Organic photosensitizers are molecules that efficiently absorb light and then transfer energy to other species. Photosensitizers are attractive for use in photochemical reactions^1–3^, energy conversion systems^4–6^, and luminophores^7–9^. However, finding a photosensitizer design that achieves both highly efficient low-energy light absorption and energy transfer remains a major challenge.

Most reported photofunctional materials with organic photosensitizers contain heavy metal atoms as effective photoactive centers. The photosensitizer undergoes efficient intersystem crossing (ISC) from the lowest singlet excited state (S_1_) to the lowest triplet excited state (T_1_) after excitation, transferring its electronic energy to an energy acceptor. According to the energy transfer (ET) process involved, the following two effective photosensitizer design strategies have been reported for luminophore photosensitization.

**Fig. 1:**
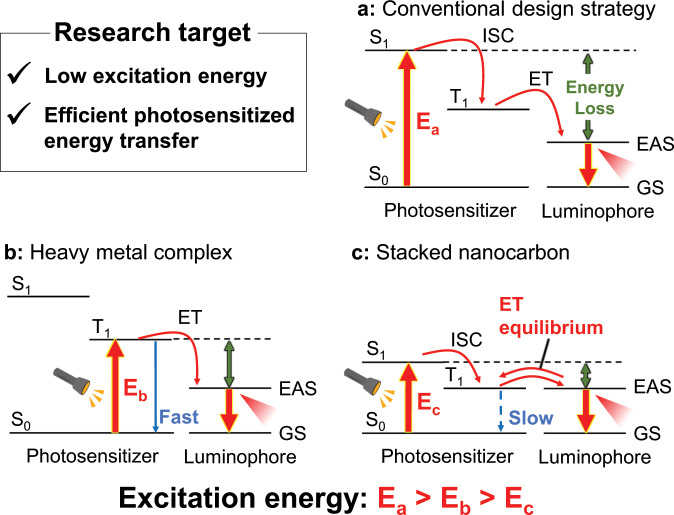
Photosensitizer design strategies. **a** Conventional design strategy for photosensitized emission (ISC: intersystem crossing, EAS: energy-accepting state, GS: ground state). **b** Heavy metal complex photosensitizer for use of low-energy light. **c** Stacked nanocarbon photosensitizer for use of low-energy light.

(a) A conventional design strategy uses strong light absorption to induce photosensitizer excitation from the ground state (S_0_) to a singlet excited state (S_n_) and tuning of an energy-donating (T_1_) level to realize effective photosensitized ET (Fig. 1a). Here, a high T_1_ level is required to suppress photon loss derived from back ET from the energy-accepting state (EAS) to T_1_. Thus, this strategy includes two energy-loss processes (ISC and ET), which makes it difficult to apply to low-energy excitation.

(b) The other major photosensitization strategy is to use a spin-forbidden transition (S-T transition) to suppress the energy-loss processes (Fig. 1b)^10–17^. The S-T transition probability can be enhanced by a heavy atom effect. For example, Ward achieved green emission from blue light-sensitized Tb(III) using photosensitizers that contained heavy metals, such as Ir(III) complexes^10^. However, this design strategy suffers from the following disadvantages. (i) The increase of S-T transition probability induced by the heavy metal effect is not large enough for the S-T transition probability to reach the spin-allowed transition probability. (ii) The heavy metal effect also increases the transition probability from T_1_ to S_0_. The rapid deactivation of T_1_ leads to ineffective ET from T_1_ to EAS. (iii) A high T_1_ level is still required to suppress photon loss derived from back ET from EAS, making it difficult to use low-energy excitation.

(c) Herein, we present a novel design to achieve photosensitized emission with low-energy excitation (Fig. 1c). The photosensitizer is based on a stacked nanocarbon composed of a large π-conjugated polyaromatic framework. The polycyclic aromatic framework was selected as a photosensitizer component because of its long T_1_ lifetime^18–21^. The long T_1_ lifetime is expected to allow the efficient use of photons even in the case of low T_1_ level with ET equilibration between EAS and T_1_. A large π-conjugated nanocarbon with high symmetry induces small Δ*E*(S_1_−T_1_)^22^, thus resulting in a high ISC yield. In addition, the stacking of the π-conjugated framework^23^ further extends the T_1_ lifetime and promotes ISC^24–27^. Thus, the stacked nanocarbon with long-lived photons and small S_1_–T_1_ and T_1_–EAS energy gaps lead to both strong low-energy light absorption and highly efficient ET.

**Fig. 2:**
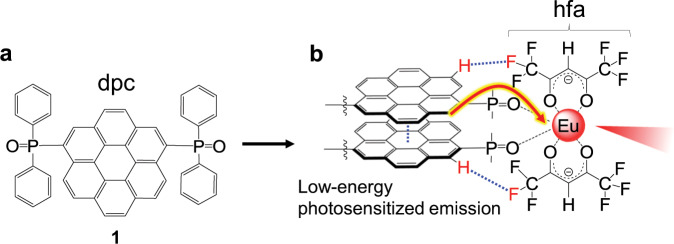
Molecular structures. **a** Chemical structures of nanocarbon ligand **1**. **b** A schematic image of Eu(III) complex containing **1**.

To demonstrate our concept, we targeted Eu(III) complexes. In Eu(III) complexes, Eu(III) and the organic ligands act as the emission center and photosensitizer, respectively. The emission lifetime of Eu(III) is usually long because it involves a forbidden 4f–4f transition^28,29^, so we expected Eu(III) to be the most appropriate acceptor to evaluate the validity of our design concept. We chose coronene as the nanocarbon antenna for the Eu(III) complex because its T_1_ level is similar to the emission energy of Eu(III). Bidentate phosphine oxide ligands, which contain the coronene framework, are introduced to the Eu(III) complex to form a rigid structure (i.e., nanocarbon ligand **1**, Fig. 2a). The rigidity of ligand **1** was an important structure factor that induced a long T_1_ lifetime^30^. To further increase the rigidity of the nanocarbon ligand, hexafluoroacetylacetonate (hfa) auxiliary ligands were used to induce formation of intramolecular CH–F interactions in the Eu(III) complex (Fig. 2b)^31^.

## Results

### Coordination structure

**Fig. 3:**
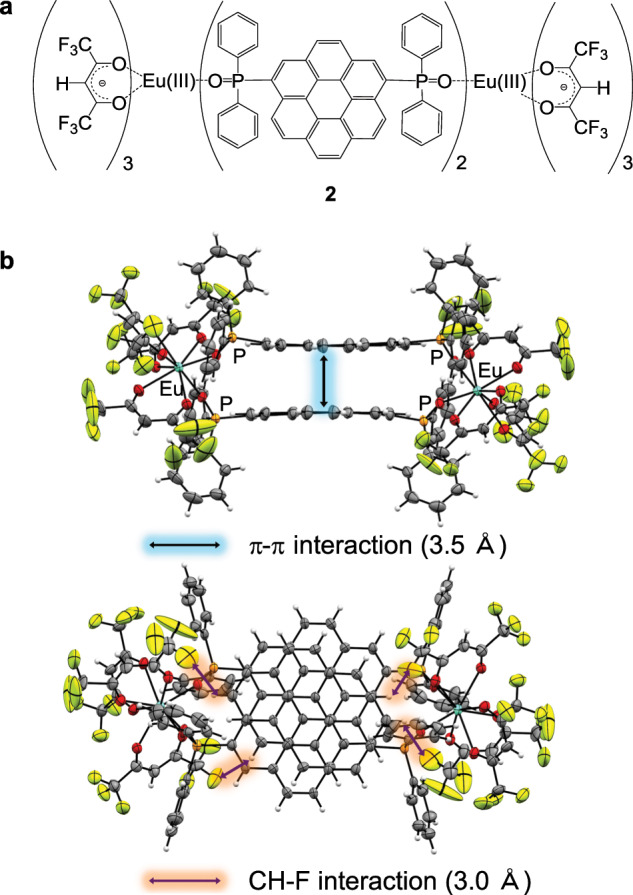
Stacked nanocarbon structure. **a** Chemical structure (**2**) and **b** ORTEP drawings (ellipsoids probability: 50%) of the Eu(III) complex.

A single crystal of the Eu(III) complex was obtained by recrystallization from a CH_2_Cl_2_/hexane solution. Single-crystal X-ray analysis revealed the formation of a dinuclear Eu(III) complex (**2**, Fig. 3, Supplementary Table 1). In the dinuclear structure, two Eu(hfa)_3_ units are connected by two nanocarbon ligands **1**. The two nanocarbon ligands are located between the Eu(III) centers and form intramolecular π–π interactions (3.5 Å), resulting in H-type exciton (Supplementary Note 1 and Supplementary Fig. 1). A shape measurement calculation^32^ indicated that the coordination geometry of Eu(III) complex **2** was an asymmetric trigonal dodecahedron (*D*_*2d*_) structure. The stacked nanocarbon ligands **1** are surrounded by hfa ligands, forming effective intramolecular CH–F interactions (3.0 Å). This structure analysis confirmed that the rigid stacked nanocarbon antenna were attached to the Eu(III) centers.

### Photophysical properties

**Fig. 4:**
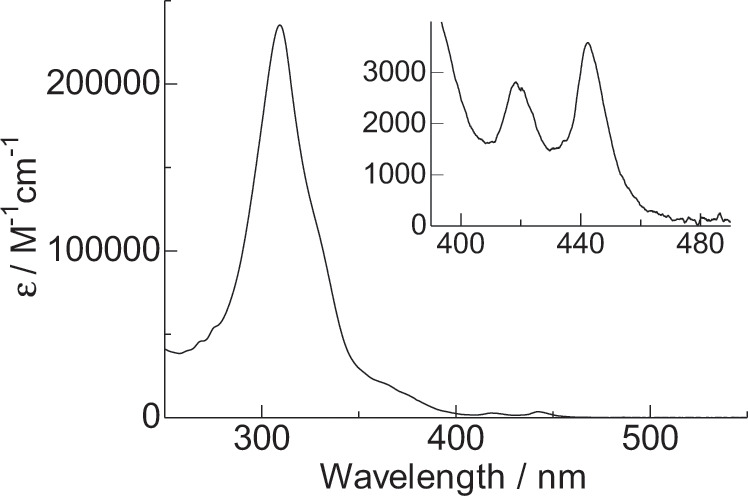
Electronic absorption spectroscopy. Electronic absorption spectrum of the Eu(III) complex (**2**) in CH_2_Cl_2_ (3.8 × 10^−6^ M).

The electronic absorption spectrum of Eu(III) complex **2** is shown in Fig. 4. Absorption bands are observed at 442 nm (3600 M^−1^ cm^−1^), 418 nm (2800 M^−1^ cm^−1^), and 309 nm (235,400 M^−1^ cm^−1^). These bands originate from π–π* transitions in the framework of nanocarbon ligand **1**. The weak and strong absorption bands at 442 and 309 nm, respectively, are attributed to the highly symmetric electronic structure with strong configuration interactions (Supplementary Note 2, Supplementary Table 2, and Supplementary Fig. 2). The absorption band at 442 nm is red-shifted from that of the free nanocarbon ligand **1** at 435 nm (Supplementary Note 3 and Supplementary Fig. 3) because of effective charge resonance interactions in complex **2**^33^. The electronic interactions induce delocalized S_n_ in the stacked nanocarbon antenna, yielding extensive absorption areas.

**Fig. 5:**
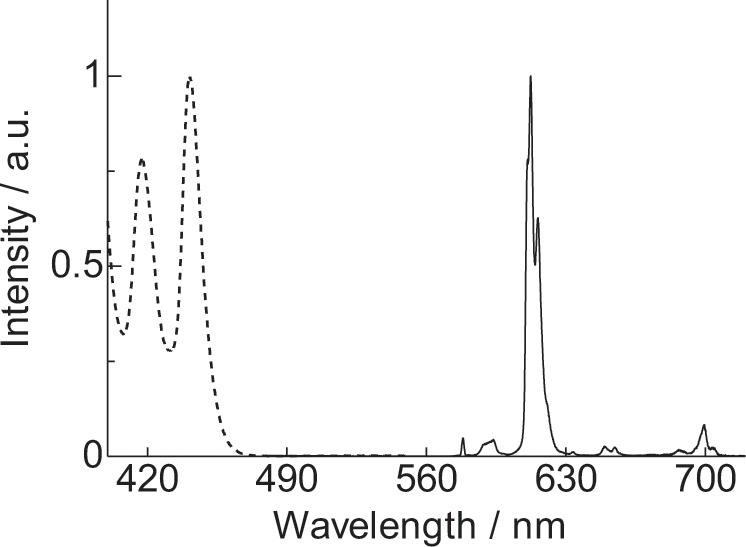
Emission properties. Emission (black solid line, *λ*_*ex*_ = 450 nm) and excitation (black broken line, *λ*_*em*_ = 613 nm) spectra of Eu(III) complex (**2**) in CH_2_Cl_2_ (3.0 × 10^−5^ M).

The emission and excitation spectra of complex **2** are presented in Fig. 5 (Supplementary Fig. 4). Emission peaks are observed at 578, 594, 612, 654, and 699 nm, which are assigned to the ^5^D_0_ → ^7^F_0_, ^5^D_0_ → ^7^F_1_, ^5^D_0_ → ^7^F_2_, ^5^D_0_ → ^7^F_3_, and ^5^D_0_ → ^7^F_4_ transitions, respectively. Time-resolved emission measurements (Supplementary Note 4 and Supplementary Figs. 5–7) of complex **2** revealed single-exponential decays with lifetimes on the scale of milliseconds (0.7 ms). The rate constants of radiative and non-radiative decay (*k*_r_ and *k*_nr_, respectively) of complex **2** calculated from the emission lifetime and spectrum^34^ are 8.8 × 10^2^ and 5.6 × 10^2^ s^−1^, respectively. The calculated emission quantum yield resulting from excitation of Eu(III) is 61%. The excitation spectrum contains peaks at 442 and 418 nm, which are consistent with peaks observed in the absorption spectrum of nanocarbon **1**, indicating effective ET from ligand **1** to Eu(III) in complex **2**.

**Fig. 6:**
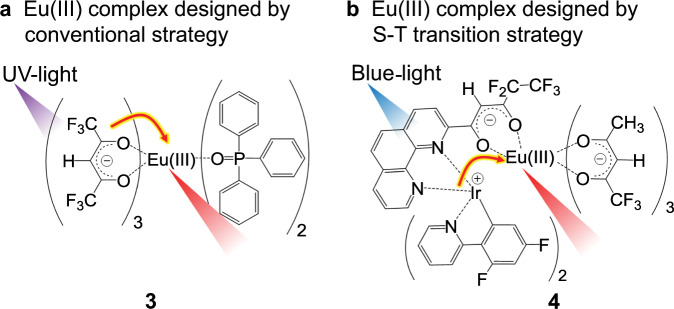
Previously reported Eu(III) complexes. **a** Eu complex **3** with UV light-sensitized emission based on the conventional design strategy (Fig. 1a)^35,36^. **b** Eu complex **4** with blue light-sensitized emission based on the S-T transition strategy (Fig. 1b)^11^.

The photosensitization properties of Eu(III) complexes fabricated based on the conventional design, heavy metal complex design, and stacked nanocarbon design strategies are compared in Table 1. Strongly luminescent Eu(III) complex **3** excited by UV light (Fig. 6a) was used as a representative molecule designed by the conventional strategy^35,36^. Luminescent Eu(III) complex **4** (Fig. 6b), which was reported to display the strongest emission when it was excited by 450-nm blue light (the lowest energy light for photosensitized Eu(III) emission)^11^ was used to represent a material designed according to the S-T transition strategy. The emission efficiency following excitation by nanocarbon ligand **1** (*Φ*_tot_) and photosensitization efficiency (*η*_sens_) of **2** are estimated to be 36% and 59%, respectively (Supplementary Note 5 and Supplementary Fig. 8). The brightness (*I*_total_) of an Ln(III) complex can be written as^37^

1 $$I_{{\mathrm{total}}} = \varepsilon \times {\mathrm{\Phi }}_{{\mathrm{tot}}}$$

**Table 1:** Photophysical properties of Eu(III) complexes (Fig. 3a (**2**), Fig. 6a, b (**3–4**))^11,35,36^.

	^a^*ε*_max_ (M^−1 ^cm^−1^)	^a^*ε*_450_ (M^−1^ cm^−1^)	^b^*ε*_tot_/%	^c^*I*_max_ (M^−1^ cm^−1^)	^c^*I*_450_ (M^−1 ^cm^−1^)
**3**^35,36^	25,200	≃0	59	1.5 × 10^4^	≃0
**4**^11^	—	600	18	—	1.1 × 10^2^
**2**	235,400	1700	36	8.5 × 10^4^	6.1 × 10^2^

^a^Molar absorption coefficient at absorption maxima (**3**: 306 nm, **2**: 309 nm) and 450 nm. The *ε*_450_ value of **4** is the molar absorption coefficient of the Ir(III)-based photosensitizer moiety at 451 nm^11^

^b^*λ*_ex_ = 370 nm (**3**), *λ*_ex_ = 480 nm (**4**), *λ*_ex_ = 450 nm (**2**)

^c^*I*_max_ = *ε*_max_ × *Φ*_tot_, *I*_450_ = *ε*_450_ × *Φ*_tot_. Here, *Φ*_totc_ is dimensionless

Based on our definition of the photosensitization performance, values of *I*_max_ (= *ε*_max_ × *Φ*_*t*ot_) and *I*_450_ (= *ε*_450nm_ × *Φ*_tot_) estimated for complex **2**–**4** are listed in Table 1. *I*_max_ of **2** (8.5 × 10^4^ M^−1^ cm^−1^) exceeds that of the strongly luminescent complex **3** (1.5 × 10^4^ M^−1^ cm^−1^). The brightness of **2** (6.1 × 10^2^ M^−1^ cm^−1^) excited by 450-nm light is five times larger than that of complex **4** (1.1 × 10^2^ M^−1^ cm^−1^), which is the Eu(III) complex with the strongest emission under 450-nm excitation reported until now (Supplementary Note 6)^11^. These results demonstrate that the stacked nanocarbon ligands induce excellent photosensitized emission properties in **2**.

### Mechanistic study

**Fig. 7:**
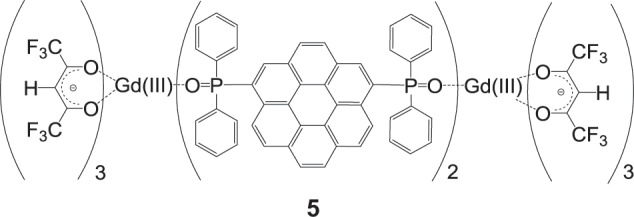
Structure of (**5**). Chemical structure of Gd(III) complex (**5**).

**Fig. 8:**
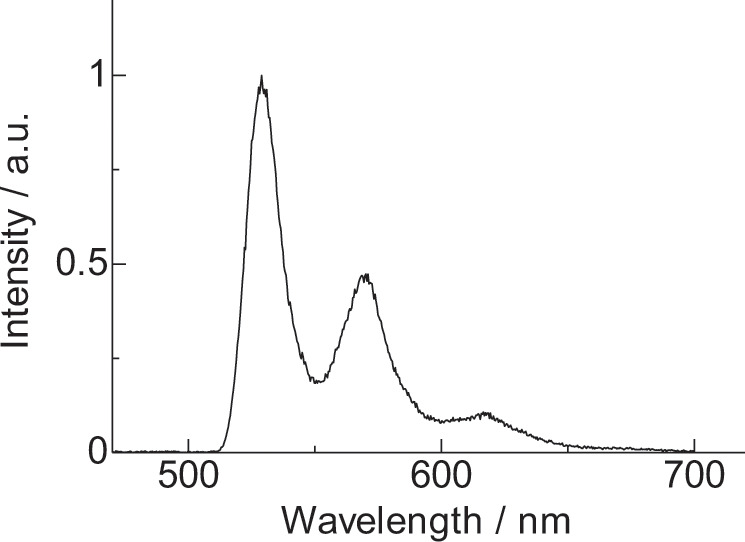
Phosphorescence spectroscopy. Phosphorescence spectrum of Gd(III) complex (**5**) (90 K, delay: 50 ms, 2Me-THF: 6.0 × 10^−5^ M, *λ*_ex_ = 420 nm).

To confirm the mechanism of the extremely high *I*_450_ of **2**, the phosphorescence spectrum of Gd(III) complex **5** (Fig. 7) in 2-methyltetrahydrofuran (6.0 × 10^−5^ M) was measured to estimate T_1_ of the stacked nanocarbon ligands **1** (Fig. 8). The estimated T_1_ level (18,900 cm^−1^) yielded a smaller Δ*E*(S_1_−T_1_) (3700 cm^−1^) than those of typical organic compounds (ex. Eu(III) complex **3**^35,36^, anthracene^38^, and a phthalocyanine derivative^39^ are 11,100, 11,700, and ca. 5000 cm^−1^, respectively) (Supplementary Fig. 9). The EAS of Eu(III) that accepts energy from the stacked nanocarbon (T_1_ = 18,900 cm^−1^) in **2** corresponds to the ^5^D_0_ level (17,250 cm^−1^) in contrast to existence of the several EAS (^5^D_0_, ^5^D_1_: 19,100 cm^−1^, ^5^D_2_: 21,400 cm^−1^) of Eu(III) complexes **3** (T_1_ = 21,700 cm^−1^, Supplementary Note 7 and Supplementary Fig. 10)^40^ and **4** (T_1_ = 21,300 cm^−1^)^11^. Although a direct energy transfer to the ^5^D_0_ level is not allowed^41^, the energy transfer from T_1_ to ^5^D_0_ in the Eu(III) complex can be induced by the J-mixing effects and thermal population of the ^7^F_1_ level. The energy gap between the photosensitizer and energy acceptor (1650 cm^−1^) induces strong back ET from ^5^D_0_ to T_1_ in **2**^42,43^.

**Fig. 9:**
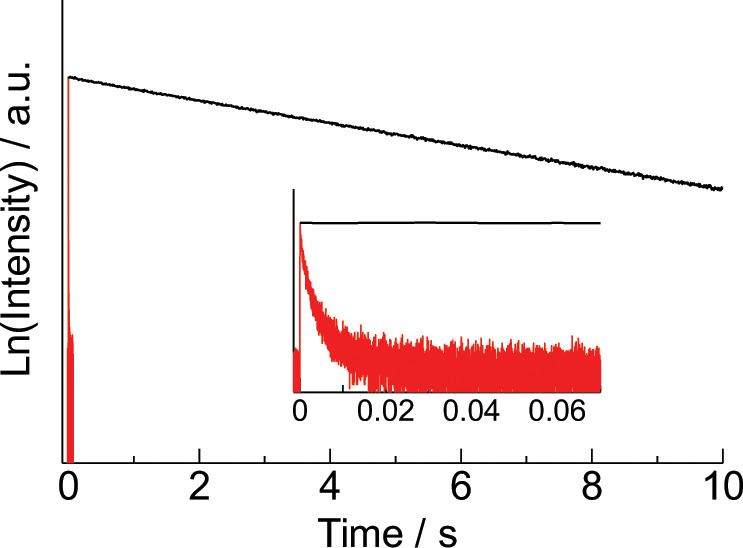
Time-resolved emission intensities. Phosphorescence decays of Gd(III) complex (**5**) (black solid line, 100 K, delay: 50 ms, 2Me-THF: 6.0 × 10^−5^ M, *λ*_ex_ = 420 nm) and previous Gd(III) complex with the same ligands of **3** (red solid line, 100 K, delay: 0 ms, solid state, *λ*_ex_ = 355 nm)^40^.

To clarify the efficient energy migration mechanism with back ET, the time-resolved emission profile of Gd(III) complex **5** was also measured (Fig. 9, black solid line). The estimated T_1_ lifetime was 6.2 s, which is 1000 times longer than those of previously reported efficient photosensitized antennas designed by conventional strategy (Fig. 9, red solid line)^40^. The long T_1_ lifetime at 100 K is considered to be based on the small radiative rate constant originating from a small spin-orbit coupling. We estimated the T_1_ lifetime of **5** at 300 K using the Arrhenius plots of temperature-dependent emission lifetime (Supplementary Note 8 and Supplementary Fig. 11)^44^. The estimated T_1_ lifetime is 40 ms, which is about 50 times longer than Eu(III) emission lifetime. This long lifetime caused by the stacked nanocarbon ligands allows efficient ET from the nanocarbon ligands to Eu(III) (T_1_ → ^5^D_0_) and strong population of the emitting ^5^D_0_ level from the low-lying T_1_ level. In contrast to our strategy, since the lifetime of Eu(III) complex (**4**) is short enough (24 μs), a high T_1_ level is required to suppress the photon loss derived from back ET from EAS (Fig. 1b).

The small Δ*E*(S_1_−T_1_) is also important for both low-energy light absorption and efficient energy migration (Fig. 1c). The light-absorbing energy level *E*(S_1_) can be expressed as:

2 $$E\left( {{\mathrm{S}}_1} \right) = E\left( {{\mathrm{T}}_1} \right) + 2K_{{\mathrm{if}}}$$

where *K* is an exchange integral between orbital pairs and subscripts i and f are the occupied and unoccupied orbitals related to S_1_, respectively. Molecules with extended π-conjugation show small Δ*E*(S_1_−T_1_) because of the small *K*_if_^24^. The efficient ET from the low *E*(T_1_) and small 2*K*_if_ enable efficient emission from **2** under low-energy light irradiation. The previously reported Eu(III) complex with high *I*_450_ (**4**) uses the S-T transition involving the heavy atomic effect (Fig. 1b) to achieve an extensive absorption area. In contrast, we developed a novel design concept based on a metal-free stacked nanocarbon ligand **1** for the achievement of small Δ*E*(S_1_−T_1_) and Δ*E*(T_1_−EAS). This strategy achieves not only an extensive absorption area but also efficient energy migration to realize a high-performance photosensitizer.

In this study, we proposed a stacked nanocarbon photosensitizer to enhance photosensitized emission efficiency. Based on the design strategy, we demonstrated that the brightness of the Eu(III) complex **2** with stacked-coronene photosensitizers exhibited 6.1 × 10^2^ M^−1^ cm^−1^ when excited by blue light, which is five times higher than the maximum brightness of a previously reported Eu(III) complex excited by 450-nm light (1.1 × 10^2^ M^−1^ cm^−1^)^11^. This study provides insights for the photosensitizer design in order to develop photofunctional materials that utilize low-energy light.

## Methods

### General method

^1^H-NMR spectra were recorded in CDCl_3_ on a JEOL ECS-400 (400 MHz) spectrometer; tetramethylsilane was used as the internal reference. Electron ionization (EI) and electrospray ionization (ESI) mass spectrometry were performed using JEOL JMS-T100 GCv and JEOL JMS-T100 LP instruments, respectively. Elemental analyses were performed using MICRO CORDER JM10. UV-vis absorption spectra for ligand **1** and Eu(III) complex **2** were measured using a JASCO V-670 spectrophotometer. Emission spectrum, excitation spectrum, and emission lifetime for Eu(III) complex **2** were measured using a Horiba FluoroLog®3 spectrofluorometer. Emission spectrum and lifetime for Gd(III) complex **5** were measured using a FP-6300 spectrofluorometer with a nitrogen bath cryostat (Oxford Instruments, Optistat DN) and a temperature controller (Oxford Instruments ITC-502S). Emission spectrum for the ligand **1** was measured using a FP-6300 spectrofluorometer with a nitrogen bath cryostat (Oxford Instruments, Optistat DN) and a temperature controller (Oxford Instruments ITC-502S). Emission quantum yield for Eu(III) complex **2** was measured using a FP-6300 spectrofluorometer with an integration sphere (ILF-533).

### Preparation of ligand 1

A solution of *n*-butyllithium (*n*-BuLi, 2.0 ml, 3.14 mmol) was added dropwise to a suspension of 1,6-dibromo-coronene (530 mg, 1.16 mmol) in dry THF (150 mL) at −80 °C under Ar. After cooling for 30 min, chlorodiphenylphosphine (0.5 mL, 2.71 mmol) was added to the suspension, which was then stirred for 7 h at room temperature. The product was evaporated and extracted using dichloromethane; the extract was washed with distilled water and then dried over anhydrous MgSO_4_. The solution was cooled and a 30% H_2_O_2_ aqueous solution (2 mL) was added. The reaction mixture was stirred for 3 h. The product was again extracted using dichloromethane; the extract was washed with distilled water and then dried over anhydrous MgSO_4_. The compounds were separated by silica gel chromatography with ethyl acetate as the mobile phase. The solvent was evaporated to yield a yellow powder (Fig. 2a).

ESI-MS: *m*/*z* calcd for C_48_H_31_O_2_P_2_ [M + H]^+^ = 701.18; found: 701.18.

IR (ATR): 3052 (st, C–H), 1182 (st, P=O) cm^−1^.

^1^H-NMR (400 MHz, CDCl_3_): δ/ppm = 7.45–7.66 (m, 12H), 7.79–7.94 (m, 8H), 8.59–8.96 (m, 8H), 9.61–9.69 (q, 1H), 9.71–9.77 (t, 1H).

### Preparation of Eu(III) complex 2

Dichloromethane (30 mL) containing Eu(hfa)_3_(H_2_O)_2_ (300 mg, 0.37 mmol) and ligand **1** (175 mg, 0.25 mmol) was refluxed under stirring for 2 h at 40 °C. The reaction mixture was filtrated, and the filtrate was concentrated using a rotary evaporator. Recrystallization from CH_2_Cl_2_/hexane solution gave yellow crystals (Yield: 3%, 25 mg, Fig. 3a).

ESI-MS: *m*/*z* calcd for C_121_H_65_Eu_2_F_30_O_14_P_4_ [M-hfa]^+^ = 2741.13; found: 2741.17.

Elemental analysis (%): calcd for C_126_H_66_Eu_2_F_36_O_16_P_4_: C 51.34, H 2.26; found: C 51.46, H 2.22.

IR (ATR): 1143 (st, P=O), 1251 (st, C-F), 1652 (st, C=O), 3061 (st, arC-H) cm^−1^.

Further information on the materials and preparation is given in the Supplementary Methods section.

### Single-crystal X-ray structure determination

X-ray crystal structures and crystallographic data for Eu(III) complex **2** is shown in Fig. 3b and Table S1. Single crystals of the compounds were mounted on micromesh (MiTeGen M3-L19-25L) using paraffin oil. Measurements were made by using a Rigaku RAXIS RAPID imaging-plate area detector or XtaLAB AFC11 (RCD3) with graphite-monochromated Mo-Kα radiation. Non-hydrogen atoms were anisotropically refined. All calculations were performed using a crystal-structure crystallographic software package. The CIF data were confirmed by the check CIF/PLATON service. CCDC-1885659 (for Eu(III) complex **2**) contain the supplementary crystallographic data for this paper. These data can be obtained free of charge from The Cambridge Crystallographic Data Centre via www.ccdc.cam.ac.uk/data_request/cif.

### Calculation of emission quantum yield

The emission quantum yields excited by Eu(III) ion (*Φ*_ff_) and the radiative (*k*_r_) and non-radiative (*k*_nr_) rate constants were estimated using equations as follows^34–36^.

3 $$\tau _{{\mathrm{rad}}} = \frac{1}{{k_{\mathrm{r}}}}$$

4 $$\tau _{{\mathrm{obs}}} = \frac{1}{{k_{\mathrm{r}} + k_{{\mathrm{nr}}}}}$$

5 $$\Phi _{{\mathrm{ff}}} = \frac{{k_{\mathrm{r}}}}{{k_{\mathrm{r}} + k_{{\mathrm{nr}}}}} = \frac{{\tau _{{\mathrm{obs}}}}}{{\tau _{{\mathrm{rad}}}}}$$

6 $$k_{\mathrm{r}} = A_{{\mathrm{MD}},0}n^3\left( {\frac{{I_{{\mathrm{tot}}}}}{{I_{{\mathrm{MD}}}}}} \right)$$

7 $$k_{{\mathrm{nr}}} = \frac{1}{{\tau _{{\mathrm{obs}}}}} - \frac{1}{{\tau _{{\mathrm{rad}}}}}$$

where *A*_MD,0_ is the spontaneous luminescence probability for the ^5^D_0_ → ^7^F_1_ transition in vacuo (14.65 s^−1^), *n* is the refractive index of the medium (1.5), and (*I*_tot_/*I*_MD_) is the ratio of the total area of the Eu(III) luminescence spectrum to the area of the ^5^D_0_ → ^7^F_1_ transition band.

## Supplementary information

Supplementary Information

Description of Supplementary Data 1

Supplementary Data 1

## Data Availability

The authors declare that the data supporting the findings of this study are available within the paper and its supplementary information. Data for the crystal structures reported in this paper have been deposited at the Cambridge Crystallographic Data Centre (CCDC) under the deposition numbers CCDC-1885659 (**2**).
