# Supplementary material for: Stacked nanocarbon photosensitizer for efficient blue light excited Eu(III) emission
Source: Commun Chem. 2020 Jan 3;3:3. doi: 10.1038/s42004-019-0251-z (PMC9812264; doi:10.1038/s42004-019-0251-z)
Supplement: Supplementary file 1 — Supplementary Information [file 42004_2019_251_MOESM1_ESM.pdf]

Stacked nanocarbon photosensitizer for efficient blue light excited Eu(III) emission

Yuichi Kitagawa*[1,3], Fumiya Suzue[1], Takayuki Nakanishi[2], Koji Fushimi[1], Tomohiro Seki[1], Hajime Ito[1,3] & Yasuchika Hasegawa*[1,3]

[1]Faculty of Engineering, Hokkaido University, Kita-13, Nishi-8, Sapporo, Hokkaido, 060-8628, Japan.

[2]Faculty of Industrial Science and Technology, Tokyo University of Science, 6-3-1, Niijuku, Katsushika-ku, Tokyo 125-8585, Japan.

[3]Institute for Chemical Reaction Design and Discovery (WPI-ICReDD), Hokkaido University, Sapporo, Hokkaido 001-0021, Japan.

Correspondence and requests for materials should be addressed to Y.K. (email: y-kitagawa@eng.hokudai.ac.jp) or Y.H. (email: hasegaway@eng.hokudai.ac.jp)

Supplementary Methods

Materials

Magnesium sulfate, anhydrous (> 98.0% (titration)), *n*-butyllithium in hexane (for organic synthesis), and chloroform-d1 (99.8%) were purchased from Kanto Chemical Co., Inc. Tetrahydrofuran (THF), super dehydrated, with a stabilizer (for organic synthesis), hydrogen peroxide (30%), bromine (Guaranteed Reagent), and dichloromethane (spectroscopic grade) were purchased from Wako Pure Chemical Industries, Ltd. Coronene (> 83.0%), chlorobenzene (> 98.0%), and chlorodiphenylphosphine (> 97.0%) were purchased from Tokyo Chemical Industry Co., Ltd.

Preparation of 1,6-dibromo-coronene

Chlorobenzene (20 ml) containing Br_2 (5.0 g, 31.3 mmol) was added dropwise to a solution of coronene (5.3 g, 17.7 mmol) in chlorobenzene (650 ml) under Ar. After stirring for 12 h at 110 °C, the solution was stirred for 3 h at RT. After decantation, the solution was filtered and washed with hot toluene and water, yielding a yellow powder.

EI-MS: m/z calcd for $C_{24}H_{10}Br_2$ $[M]^+$ = 457.91; found: 457.87.

Preparation of Gd(III) complex (Fig. 7, **5**)

Dichloromethane (30 mL) containing $Gd(hfa)_3(H_2O)_2$ (100 mg, 0.12 mmol) and ligand **1** (40 mg, 0.057 mmol) was refluxed under stirring for 2 h at 40 °C. The reaction mixture was filtrated, and the filtrate was concentrated using a rotary evaporator. Recrystallization from CH_2Cl_2/hexane solution gave yellow crystals (Yield: 3 %, 5.0 mg).

ESI-MS: m/z calcd for $C_{121}H_{65}Gd_2F_{30}O_{14}P_4$ $[M\text{-}hfa]^+$ = 2751.13; found: 2751.11.

Elemental analysis (%): calcd for $C_{126}H_{66}Gd_2F_{36}O_{16}P_4$+$CH_2Cl_2$: C 50.13, H 2.25. found: C 49.72, H 2.01.

Supplementary Table 1. Crystal data of Eu(III) complex (**2**)

	Eu(III) complex (**2**)
chemical formula	$C_{126}H_{66}Eu_2F_{36}O_{16}P_4$
formula weight	2947.61
crystal color, habit	yellow, needle
crystal system	monoclinic
space group	$P2_1/n$
a / Å	13.5541(6)
b / Å	25.6188(10)
c / Å	18.0509(8)
α / deg	90
β / deg	95.759(7)
γ / deg	90
volume / Å	6236.3(5)
Z	2
density / g cm^{-3}	1.570
Temperature / °C	−150
μ (Mo K$_\alpha$) / cm^{-1}	12.22
max 2θ / deg	54.972
Reflections	14305
R_1	0.0698
wR_2	0.2023

Supplementary Note 1 (Structural analysis)

The continuous shape measure factor S was calculated to estimate distortion degree of the coordination structure in the first coordination sphere based on the crystal structure data. The S value is given by following Supplementary Equation 1.[1]

$$S = \min \frac{\sum_k^N |Q_k - P_k|^2}{\sum_k^N |Q_k - Q_o|^2} \times 100 \qquad (1),$$

where Q_k is the vertices of an actual structure, Q_0 is the center of mass of an actual structure, N is the number of vertices and P_k is the vertices of an ideal structure. The 8-coordinated lanthanide complexes exhibit square antiprism (SAP, point group: D_{4d}), trigonal dodecahedron (TDH, point group: D_{2d}), and biaugmented trigonal prism (BTP, point group: C_{2v}) structures, according to evaluation of the S values. From the calculation, complex **2** is categorized to be asymmetric coordination geometry of TDH (D_{2d}) structure (S(SAP) = 1.37, S(TDH) = 0.56, S(BTP) = 1.54).

The exciton coupling strength between two molecules is expressed as Supplementary Equation 2[2]:

$$V = \frac{|\mathbf{M}|^2}{|\mathbf{r}|^3}(\cos\alpha - 3\cos\theta_1 \cdot \cos\theta_2) \qquad (2),$$

where **M** is the transition dipole moment of the monomer. The other parameters are expressed in Supplementary Figure 1. We calculated the exciton coupling strength between two ligands (**1**) using the orientation parameter based on the X-ray crystal structure (Fig. 3b). The calculated exciton coupling strength is positive ($0.87|\mathbf{M}|^2/|\mathbf{r}|^3 > 0$), indicating the formation of an H-type orientation.

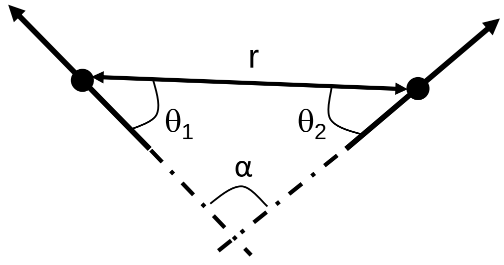

Supplementary Figure 1. Oblique arrangement of two transition dipole moments.

Supplementary Note 2 (DFT calculation of ligand 1)

To clarify the electronic properties of ligand **1**, its electronic absorption spectrum was calculated using the time-dependent density functional theory method with the B3LYP/6-31G(d, p) basis set using the structure of the ligand moiety identified in the single-crystal X-ray data. The characteristic weak absorption bands at longer wavelength and strong absorption bands at shorter wavelength (Supplementary Figure 3, black line) were reproduced in the calculated spectrum (Supplementary Table 2). The origins of these bands can be qualitatively explained by configuration interactions involving four π molecular orbitals (HOMO−1, HOMO, LUMO, and LUMO+1, Supplementary Figure 2; HOMO is highest occupied molecular orbital and LUMO is lowest unoccupied molecular orbital) and four electronic configurations ((HOMO−1 → LUMO), (HOMO−1 → LUMO+1), (HOMO→LUMO), and (HOMO→LUMO+1)). Because of the small energy difference between HOMO and HOMO−1, a heavy admixture forms between the electronic configurations composed of these orbitals, resulting in both the intense high-energy bands and weak low-energy bands.

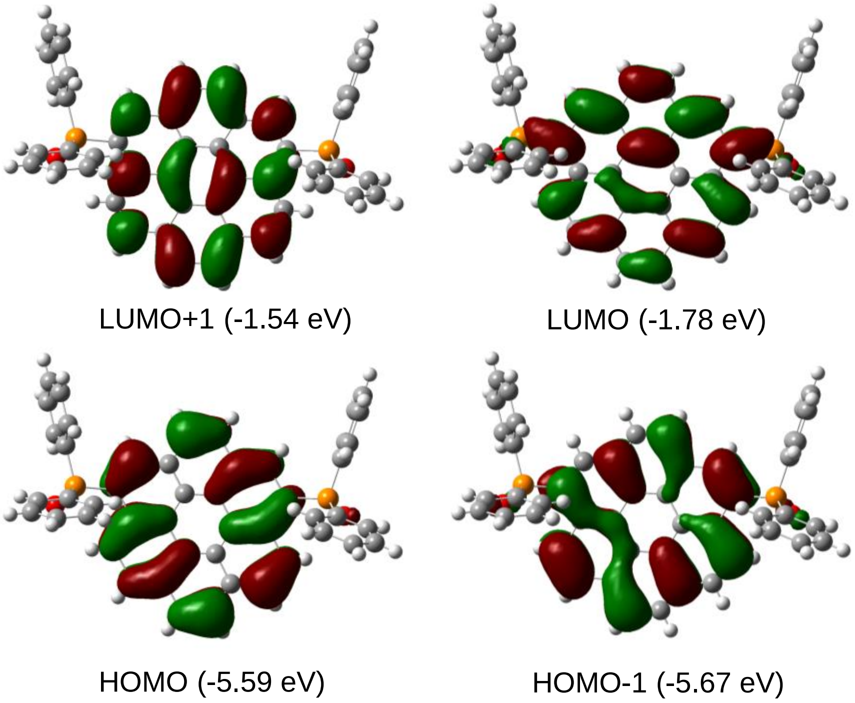

Supplementary Figure 2. Molecular orbitals of ligand **1**.

Supplementary Table 2. Information about the main electronic configurations in the absorption bands.

	λ_{abs}/nm	f	main contribution
S_1	391	0.0096	19%(HOMO-1→LUMO), 23%(HOMO-1→LUMO+1), 44%(HOMO→LUMO), 14%(HOMO→LUMO+1)
S_2	370	0.0580	47%(HOMO-1→LUMO), 8%(HOMO-1→LUMO+1), 24%(HOMO→LUMO), 20%(HOMO→LUMO+1)
S_3	316	0.7632	26%(HOMO-1→LUMO), 56%(HOMO→LUMO+1)
S_4	310	0.4108	54%(HOMO-1→LUMO+1), 26%(HOMO→LUMO)

Supplementary Note 3 (Experimental data of ligand 1)

Electronic absorption and phosphorescence spectra of the ligand **1** are shown in Supplementary Figure 3. The T_1 level of Eu(III) complex **2** (Fig. 8, 18,900 cm^{-1}) was red-shifted from that of the free nanocarbon ligand **1** (Supplementary Figure 3, 19,100 cm^{-1}), thus delocalizing T_1 of the integrated photosensitized antenna through charge-resonance interactions.

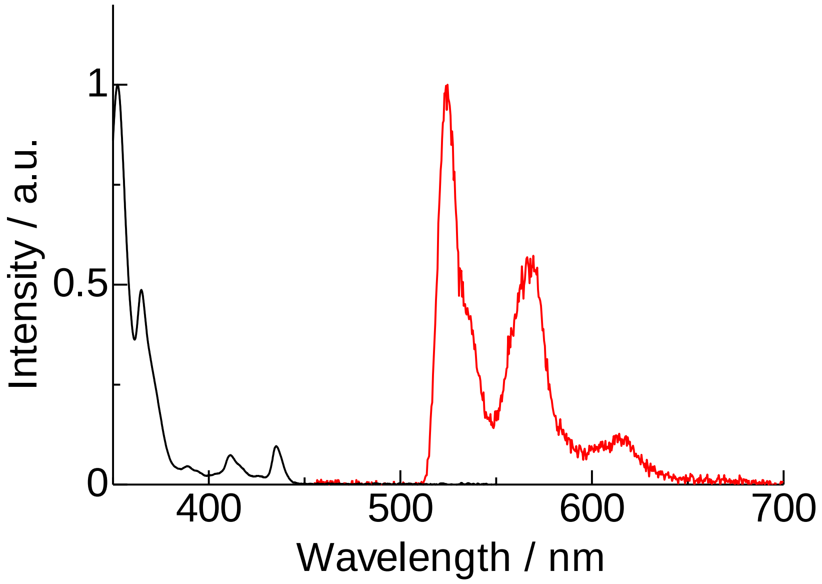

Supplementary Figure 3. Electronic absorption (black line, 1.4 × 10^{-5} M, CH_2Cl_2) and phosphorescence (red line, 2.0 × 10^{-5} M, 2Me-THF, 90 K, delay: 50 ms, λ_{ex} = 430 nm) spectra of the ligand **1**.

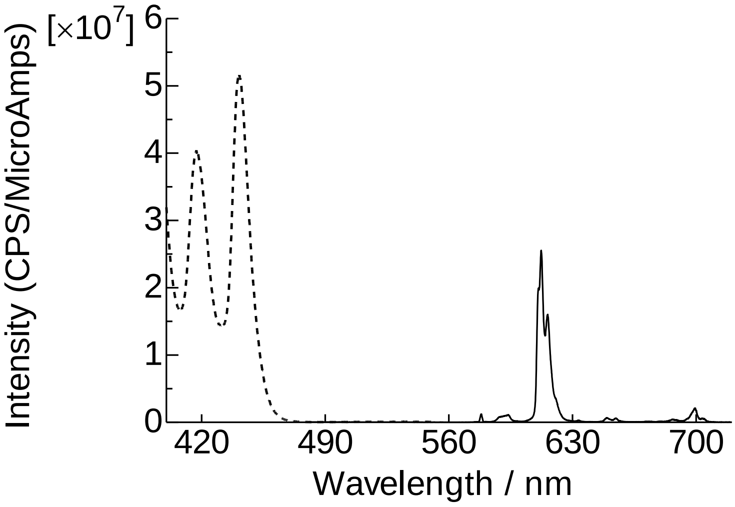

Supplementary Figure 4. Emission (black solid line, λ_{ex} = 450 nm) and excitation (black broken line, λ_{em} = 613 nm) spectra of Eu(III) complex (**2**) in CH_2Cl_2 (3.0×10^{-5} M).

Supplementary Note 4 (Excited state dynamics)

Energy transfer from the triplet state of an organic ligand to a lanthanide ion becomes reversible when the triplet state can be thermally repopulated from the lanthanide-centered excited state, resulting in an excited state equilibrium. The lifetime measurement dependence on the oxygen concentration for the lanthanide complex is a useful method for confirming the existence of the excited state equilibrium between the T_1 state and lanthanide-centered excited state.[3-4] This can be attributed to the existence of the T_1 and 4f-4f excited states that are sensitive and insensitive toward O_2, respectively. The existence of the excited state equilibrium manifests the dependence of the lanthanide emission lifetime (time-dependent concentration of excited $^5D_0$) on the oxygen concentration, which involves a deactivation process from the T_1 state upon reaction with oxygen molecules (Supplementary Figure 5).

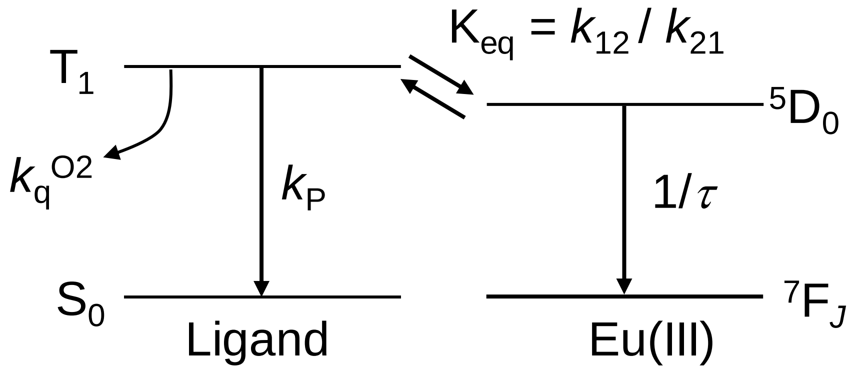

Supplementary Figure 5. Schematic energy levels for the equilibrium processes in an Eu(III) complex and the effect of dissolved oxygen. k_{12}: Forward energy transfer rate constant, k_{21}: back energy transfer rate constant, $k_{21} = k_{12} \exp(-\Delta E/RT)$.[3]

The emission decay curves of Eu(III) complex (**2**) and (**3**) in non-degassed CH_2Cl_2 and degassed CH_2Cl_2 are shown in Supplementary Figure 6. The emission lifetime of Eu(III) complex (**2**) in degassed CH_2Cl_2 (0.7 ms) is longer than that in non-degassed CH_2Cl_2 (0.5 ms), which is caused by T_1 quenching by oxygen. The results reveal the existence of equilibrium between the excited state (T_1) and the emitting level ($^5D_0$) of Eu(III) ion. The emission lifetime of Eu(III) complex **3** (Supplementary Figure 7) in degassed CH_2Cl_2 (0.82 ms) is almost the same as that in non-degassed CH_2Cl_2 (0.81 ms), which reveals the nonexistence of equilibration between the excited state (T_1) and the emitting level ($^5D_0$) of the Eu(III) ion. The non-emissive phosphorescence of the ligand moiety of the Eu(III) complex (**2**) during equilibration might be due to a very slow radiative rate constant (<

0.2 s^{-1}) from the T_1 state of stacked nanocarbon.

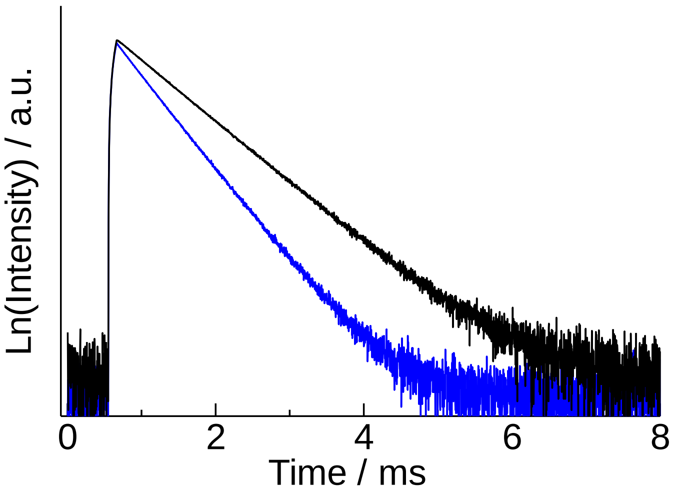

Supplementary Figure 6. Emission decay curves of Eu(III) complex **2** in the degassed (black line) and the non-degassed CH_2Cl_2 solution (blue line) (3.0×10^{-5} M, λ_{ex} = 356 nm, λ_{em} = 613 nm).

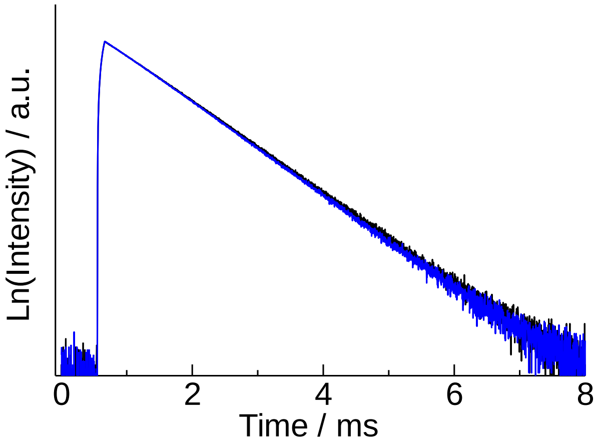

Supplementary Figure 7. Emission decay curves of Eu(III) complex **3** in the degassed (black line) and the non-degassed CH_2Cl_2 solution (blue line) (1.0×10^{-3} M, λ_{ex} = 356 nm, λ_{em} = 613 nm).

Supplementary Note 5 (Excitation spectrum of Eu(III) complex (2))

Excitation spectrum of Eu(III) complex (**2**) is shown in Supplementary Figure 8. The spectrum is similar to the absorption spectrum, and shows the characteristic strong band at around 310 nm and weak bands at 420 nm and 440 nm. The absorption peak intensity ratio (Abs_{310nm}/Abs_{420nm} = 89) is comparable with the excitation peak intensity ratio (Ex_{310nm}/Ex_{420nm} = 96), which indicates that the excitation wavelength-dependent emission quantum yields are small.

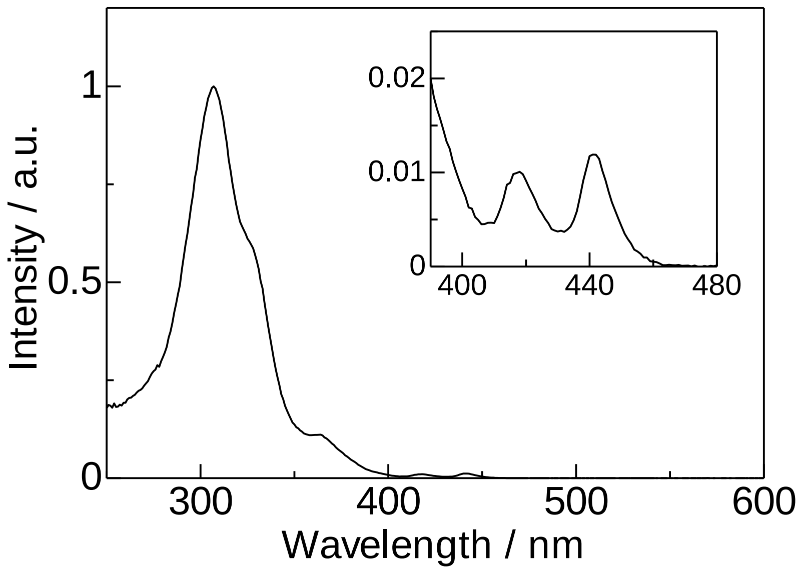

Supplementary Figure 8. Excitation spectrum of Eu(III) complex (**2**) in dilute solution (CH_2Cl_2, 1.0 × 10^{-6} M, λ_{em} = 613 nm).

Supplementary Note 6 (Eu(III) emission excited by a blue light)

The development of red phosphors excited by a blue LED is required to fabricate high-efficiency white LEDs. Recently, the efficient red luminescence of a brick-type Eu(III) complex excited by a blue LED was achieved for the first time.[5] I_{450} of Eu(III) complex **2** (5.5×10^2 $M^{-1}cm^{-1}$) is much larger than that of the previous brick-type Eu(III) complex (0.9×10^2 M^{-1} cm^{-1}). Although several blue-light excitable Eu(III) complexes in solid state (high concentration states to compensate a very weak absorption coefficient at 450 nm) have been reported[6], the reports on blue-light excitable Eu(III) complexes in dilute solutions are very few.[7] To the best of our knowledge, only two Eu(III) complexes (**2** and **4**) showing effective Eu(III) emission and long absorption peak wavelengths (> 440 nm) are reported till date.

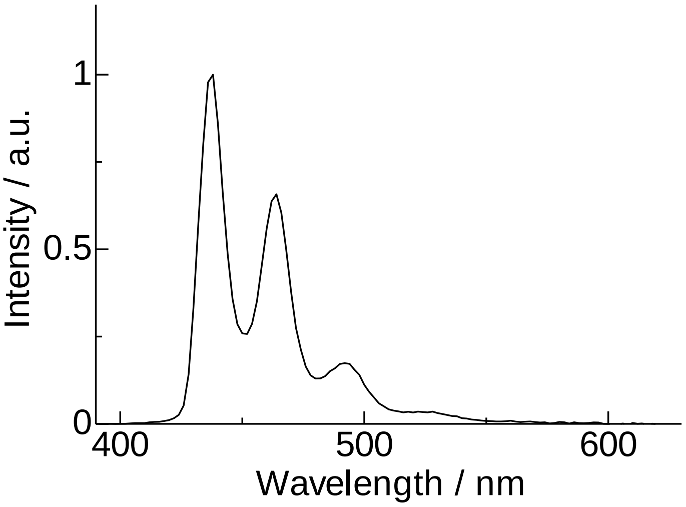

Supplementary Figure 9. Fluorescence spectrum of Gd(III) complex (**5**) in 2Me-THF (8.0×10^{-6} M, λ_{ex} = 350 nm).

Supplementary Note 7 (Energy transfer analysis using LUMPAC)

To obtain a deeper understanding of excited state dynamics for complexes **3** and **2**, we calculated the energy transfer rates between ligands and Eu(III) ions using LUMPAC software.[8] The ground-state coordination geometries of the Eu(III) complexes **2** and **3** as isolated molecules (vacuum condition) were calculated using the Sparkle/RM1 model. We used the optimized structure of complex **2** and **3** to estimate the singlet and triplet excited states with the configuration interaction singles (CIS) method based on the intermediate neglect of differential overlap/spectroscopic (INDO/S) technique, as proposed in previous reports.[8-13] The calculations were performed using ORCA software (v.4.2.0).[14] We used a point charge (+3) to represent the trivalent europium ion. The S_1 energies of **3** and **2** were estimated to be 31,500 and 25,700 cm^{-1}, respectively. The T_1 energies of complexes **3** and **2** were estimated to be 20,100 and 18,900 cm^{-1}, respectively. Although the calculated T_1 energies of **3** were underestimated, they were similar to the experimental values (**3**: 21,700 cm^{-1}, **2**: 18,900 cm^{-1}). Using the electronic structures, we calculated the total emission quantum yields and rate constants using LUMPAC. For complex **3**, the calculated emission quantum yield (68%) closely resembles the experimental value (59%), which indicates the validity of the calculation. The calculated Judd-Ofelt parameters (Ω_2: 28.07 $\times 10^{-20}$ cm^{-1}, Ω_4: 6.49 $\times 10^{-20}$ cm^{-1}) were also similar

with the experimental results (Ω_2: 29.81 $\times 10^{-20}$ cm^{-1}, Ω_4: 7.23 $\times 10^{-20}$ cm^{-1}). The rate constants for the excited states of **3** are shown in Supplementary Figure 10. The energy transfer rates from S_1 to $^5D_4$ and $^5D_4$ to S_1 are 2.4 $\times$ 10^3 and 3.0 $\times$ 10^{-15} s^{-1}, respectively. We also calculated the energy transfer rates from singlet states using the singlet energy calculated by DFT calculation at the B3LYP level (6-31G(d, p)).[15-16] The S_1 energy is estimated to be 29500 cm^{-1}, and the calculated energy transfer rates from singlet state to $^5D_4$ and $^5D_4$ to singlet state are 2.8 $\times$ 10^4 and 7.8 $\times$ 10^{-9} s^{-1}, respectively. The results indicate that energy transfers from S_1 state are ineffective. On the other hand, the energy transfer rates from T_1 to $^5D_1$, T_1 to $^5D_0$, $^5D_1$ to T_1, and $^5D_0$ to T_1 are 3.1 $\times$ 10^6, 3.9 $\times$ 10^6 s^{-1}, 2.1 $\times$ 10^4, and 6.0 s^{-1}, respectively. The results indicate that energy transfers from T_1 state are effective. These calculations also indicate that the back energy transfer from the Eu(III) ion to ligands is suppressed by the high T_1 energy level (Fig. 1a). On the other hand, the calculated emission quantum yield of **2** is very low (under 0.01%), which is in contradiction with the experimentally obtained value of 36%. The calculated Judd-Ofelt parameters (Ω_2: 30.27 $\times 10^{-20}$ cm^{-1}, Ω_4: 7.98 $\times 10^{-20}$ cm^{-1}) is similar with the experimental results (Ω_2: 30.41 $\times 10^{-20}$ cm^{-1}, Ω_4: 7.55 $\times 10^{-20}$ cm^{-1}). The disparity in emission quantum yields likely results from the very slow energy transfer rates calculated for this complex ($T_1 \rightarrow {}^5D_1$: 2.4 $\times$ 10^{-1} s^{-1}, and $T_1 \rightarrow {}^5D_0$: 3.9 $\times$ 10^{-1} s^{-1}). The inaccuracy of this result may be

due to difficulties in precisely calculating the characteristic electronic structure of the phosphine oxide ligand containing the coronene framework as an energy donor. The electronic interactions between the coronene framework and the phosphine oxide moiety (P=O) are complex. In addition, the ligands containing coronene show a relatively high HOMO energy level (DFT calculation: -5.59 eV; Supplementary Figure 2), which induces the formation of an LMCT band with a relatively low energy level. The formation of an LMCT band with low energy (ca. 25,000 cm^{-1}) has been shown to affect the electronic character of 4f-4f excited states.[17] We propose that the LMCT perturbation might account for some of the inaccuracy in the calculations.

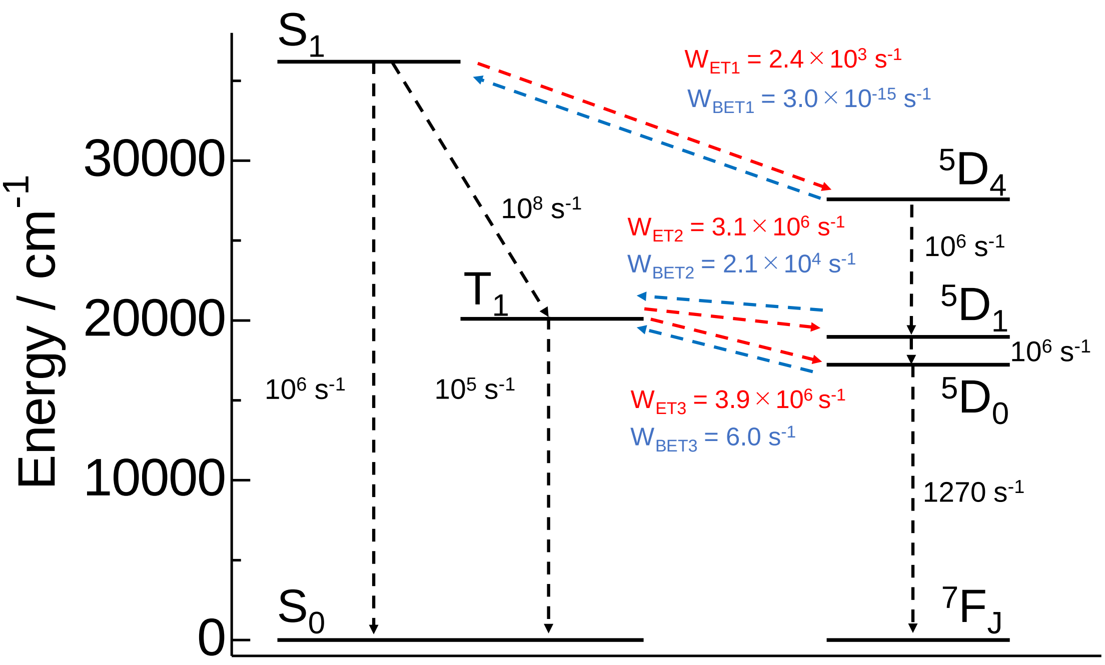

Supplementary Figure 10. Energy level diagram for complex **3** showing the energy transfer processes (W_{ET1}: $S_1 \rightarrow {}^5D_4$, W_{ET2}: $T_1 \rightarrow {}^5D_1$, W_{ET3}: $T_1 \rightarrow {}^5D_0$, W_{BET1}: $S_1 \leftarrow {}^5D_4$, W_{BET2}: $T_1 \leftarrow {}^5D_1$, W_{BET3}: $T_1 \leftarrow {}^5D_0$).

Supplementary Note 8 (Temperature dependent emission lifetime of 5)

Temperature-dependent emission lifetimes were measured for Gd(III) complex (**5**) in 2-Me-THF. We used Arrhenius plots of these measurements to estimate the T_1 lifetime of **5** at 300 K (Supplementary Figure 11, 145 K-163 K) as reported.[18] We have assumed that the decay rate does not change below 100 K (τ = 6.2 s), as a similar decay rate is observed at 100 K and 110 K. The estimated T_1 lifetime at 300 K is 40 ms, which is approximately 50 times longer than the Eu(III) emission lifetime.

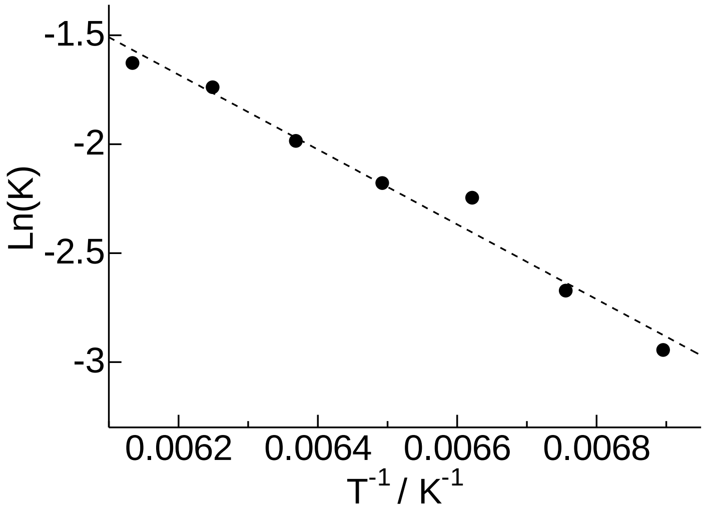

Supplementary Figure 11. Arrhenius plots of Gd(III) complex (**5**).

Supplementary References

1. Casanova, D., Llunell, M., Alemany, P. & Alvarez, S. The rich stereochemistry of eight-vertex polyhedra: a continuous shape measures study. *Chem. Eur. J.* **11**, 1479–1494 (2005).

2. Osuka, A., Maruyama, K. Synthesis of naphthalene-bridged porphyrin dimers and their orientation-dependent exciton coupling. *J. Am. Chem. Soc.* **110**, 4454–4456 (1988).

3. Quici, S., Cavazzini, M., Marzanni, G., Accorsi, G., Armaroli, N., Ventura, B., Barigelletti, F. Visible and near-infrared intense luminescence from water-soluble lanthanide [Tb(III), Eu(III), Sm(III), Dy(III), Pr(III), Ho(III), Yb(III), Nd(III), Er(III)] complexes. *Inorg. Chem.* **44**, 529–537 (2005).

4. Sørensen, T. J.; Kenwright, A. M.; Faulkner, S. Bimetallic lanthanide complexes that display a ratiometric response to oxygen concentrations. *Chem. Sci.* **6**, 2054–2059 (2015).

5. Chen, F. F., Bian, Z. Q., Liu, Z. W., Nie, D. B., Chen, Z. Q. & Huang, C. H. Highly efficient sensitized red emission from europium (III) in Ir-Eu bimetallic complexes by ^{3}MLCT energy transfer. *Inorg. Chem.* **47**, 2507–2513 (2008).

6. Koizuka, T. et al. Y. Red luminescent Eu(III) coordination bricks excited on blue LED chip. *Inorg. Chem.* **57**, 7097–7103 (2018).

7. Reddy, M. L. P.; Divya, V.; Pavithran, R. Visible-light sensitized luminescent europium(iii)-β-diketonate complexes: bioprobes for cellular imaging. *Dalton Trans.* **2013**, *42*, 15249–15262.

8. Dutra, J. D. L.; Bispo, T. D.; Freire, R. O. LUMPAC Lanthanide Luminescence Software: Efficient and User Friendly. *J. Comput. Chem.* **2014**, *35*, 772−775.

9. Ridley, J. E.; Zerner, M. C. Triplet States via Intermediate Neglect of Differential Overlap: Benzene, Pyridine and the Diazines. *Theor. Chim. Acta.* **1976**, *42*, 223−236.

10. Andres, J.; Chauvin, A.-S. Energy Transfer in Coumarin sensitised Lanthanide Luminescence: Investigation of the Nature of the Sensitiser and its Distance to the Lanthanide Ion. *Phys. Chem. Chem. Phys.* **2013**, *15*, 15981−15994.

11. Santos, J. G.; Dutra, J. D. L.; Junior, S. A.; Freire, R. O.; Junio, N. B. da C. Theoretical Spectroscopic Study of Europium Tris(bipyridine) Cryptates. *J. Phys. Chem. A* **2012**, *116*, 4318−4322.

12. Ilmi, R.; Haque, A.; Khan, M. S.; Synthesis and Photo-physics of Red Emitting Europium Complexes: An Estimation of the Role of Ancillary Ligand By Chemical Partition of Radiative Decay Rate. *J. Photochem. Photobiol., A: Chem.* **2019**, *370*, 135−144.

13. Bosshard, G. Z.; Brito, G. A.; Monteiro, J. H. S. K.; de Bettencourt-Dias, A.; Mazali, I. O.; Sigoli, F. A. Photophysical Properties of Asymmetric and Water-Soluble Dinuclear Lanthanide Complexes of Poly Glycol Chain Functionalized-Benzoic Acid Derivative: Experimental and Theoretical Approaches. *RSC Adv.* **2016**, *6*, 101133−101141.

14. Neese, F. The ORCA program system. *Comput. Mol. Sci.* **2012**, *2*, 73−78.

15. Lee, C.; Yang, W.; Parr, R. G. Development of the Colle-Salvetti Correlation-energy Formula into a Functional of the Electron Density. *Phys. Rev. B* **1988**, 37, 785.

16. Becke, A. D. Density-Functional Exchange-Energy Approximation with Correct Asymptotic Behavior. *Phys. Rev. A* **1988**, 38, 3098.

17. Yanagisawa, K.; Nakanishi, T.; Kitagawa Y.; Seki, T.; Akama T.; Kobayashi, M.; Taketsugu, T.; Ito, H.; Fushimi, K.; Hasegawa, Y. Enhanced luminescence of asymmetrical seven-coordinate Eu^{III} complexes including LMCT perturbation. *Eur. J. Inorg. Chem.* **2017**, *2017*, 3843−3848.

18. Monti, S., Orlandi, G., Kellmann, A., Tfibel, F. Temperature dependence of the phosphorescence lifetime of nitrobenzopyrans: The role of the lowest triplet state in the reaction of pyran ring opening. *J. Photochem.* **33**, 81–87 (1986).
