# Supplementary material for: Stacked nanocarbon photosensitizer for efficient blue light excited Eu(III) emission
Source: Commun Chem. 2020 Jan 3;3:3. doi: 10.1038/s42004-019-0251-z (PMC9812264; doi:10.1038/s42004-019-0251-z)
Supplement: Supplementary file 2 — Description of Supplementary Data 1 [file 42004_2019_251_MOESM2_ESM.pdf]

Description of Additional Supplementary Files

File Name: Supplementary Data 1

Description: Crystallographic data in CIF format for Eu(III) complex **2**.
